# Supplementary material for: Trophic niche differentiation and utilisation of food resources in Collembola is altered by rainforest conversion to plantation systems
Source: PeerJ. 2021 Mar 2;9:e10971. doi: 10.7717/peerj.10971 (PMC7934680; doi:10.7717/peerj.10971)
Supplement: Supplemental Information 1 [file peerj-09-10971-s001.docx]

**Supplementary Material**

**Table S1. Density, species richness and diversity indexes of Collembola in each sample**

| **Landscape** | **System** | **Plot** | **Layer** | **Year** | **Density**  **(ind m^-2^)** | **Richness  (spp. sample^-1^)** | **shannon** | **simpson** | **invsimpson** |
| --- | --- | --- | --- | --- | --- | --- | --- | --- | --- |
| Bukit Duabelas | Jungle Rubber | BJ2b | Litter | 2013 | 2613 | 8 | 1,72 | 0,78 | 4,73 |
| Bukit Duabelas | Jungle Rubber | BJ3b | Litter | 2013 | 5265 | 5 | 0,95 | 0,45 | 1,82 |
| Bukit Duabelas | Jungle Rubber | BJ4b | Litter | 2013 | 1443 | 2 | 0,68 | 0,49 | 1,96 |
| Bukit Duabelas | Jungle Rubber | BJ5b | Litter | 2013 | 975 | 4 | 0,97 | 0,57 | 2,34 |
| Bukit Duabelas | Oil Palm | BO2b | Litter | 2013 | 273 | 4 | 1,27 | 0,69 | 3,26 |
| Bukit Duabelas | Oil Palm | BO3b | Litter | 2013 | 2223 | 8 | 1,44 | 0,64 | 2,81 |
| Bukit Duabelas | Oil Palm | BO4b | Litter | 2013 | 312 | 4 | 1,25 | 0,68 | 3,20 |
| Bukit Duabelas | Oil Palm | BO5b | Litter | 2013 | 1131 | 6 | 1,60 | 0,75 | 4,10 |
| Bukit Duabelas | Rainforest | BF1b | Litter | 2013 | 2145 | 12 | 1,93 | 0,79 | 4,98 |
| Bukit Duabelas | Rainforest | BF2b | Litter | 2013 | 2106 | 5 | 1,02 | 0,57 | 2,36 |
| Bukit Duabelas | Rainforest | BF3b | Litter | 2013 | 5265 | 16 | 2,21 | 0,81 | 5,44 |
| Bukit Duabelas | Rainforest | BF4b | Litter | 2013 | 1833 | 9 | 1,86 | 0,80 | 5,24 |
| Bukit Duabelas | Rubber | BR1b | Litter | 2013 | 1794 | 7 | 1,59 | 0,76 | 4,18 |
| Bukit Duabelas | Rubber | BR2b | Litter | 2013 | 624 | 3 | 1,04 | 0,63 | 2,72 |
| Bukit Duabelas | Rubber | BR3b | Litter | 2013 | 2496 | 9 | 1,94 | 0,83 | 5,88 |
| Harapan | Jungle Rubber | HJ1b | Litter | 2013 | 6591 | 8 | 1,15 | 0,60 | 2,53 |
| Harapan | Jungle Rubber | HJ2b | Litter | 2013 | 3510 | 9 | 1,70 | 0,77 | 4,49 |
| Harapan | Jungle Rubber | HJ3b | Litter | 2013 | 156 | 4 | 1,38 | 0,75 | 4,00 |
| Harapan | Jungle Rubber | HJ4b | Litter | 2013 | 1755 | 6 | 1,41 | 0,71 | 3,55 |
| Harapan | Oil Palm | HO1b | Litter | 2013 | 1287 | 5 | 0,87 | 0,40 | 1,69 |
| Harapan | Oil Palm | HO2b | Litter | 2013 | 78 | 2 | 0,69 | 0,5 | 2,00 |
| Harapan | Oil Palm | HO3b | Litter | 2013 | 156 | 4 | 1,38 | 0,75 | 4,00 |
| Harapan | Rainforest | HF1b | Litter | 2013 | 3471 | 8 | 2,00 | 0,85 | 6,95 |
| Harapan | Rainforest | HF2b | Litter | 2013 | 39 | 1 | 0 | 0 | 1,00 |
| Harapan | Rainforest | HF3b | Litter | 2013 | 2379 | 6 | 1,53 | 0,75 | 4,03 |
| Harapan | Rainforest | HF4b | Litter | 2013 | 3237 | 9 | 1,41 | 0,64 | 2,81 |
| Harapan | Rubber | HR1b | Litter | 2013 | 3666 | 5 | 1,20 | 0,65 | 2,86 |
| Harapan | Rubber | HR2b | Litter | 2013 | 2262 | 7 | 1,60 | 0,76 | 4,19 |
| Harapan | Rubber | HR3b | Litter | 2013 | 507 | 4 | 1,03 | 0,55 | 2,25 |
| Harapan | Rubber | HR4b | Litter | 2013 | 351 | 4 | 1,27 | 0,69 | 3,24 |
| Bukit Duabelas | Jungle Rubber | BJ2b | Soil | 2013 | 1443 | 6 | 1,20 | 0,61 | 2,57 |
| Bukit Duabelas | Jungle Rubber | BJ3b | Soil | 2013 | 2067 | 7 | 1,63 | 0,77 | 4,53 |
| Bukit Duabelas | Jungle Rubber | BJ4b | Soil | 2013 | 1521 | 4 | 0,64 | 0,31 | 1,45 |
| Bukit Duabelas | Jungle Rubber | BJ5b | Soil | 2013 | 1365 | 6 | 0,94 | 0,43 | 1,75 |
| Bukit Duabelas | Oil Palm | BO2b | Soil | 2013 | 3159 | 9 | 1,41 | 0,62 | 2,66 |
| Bukit Duabelas | Oil Palm | BO3b | Soil | 2013 | 2028 | 7 | 1,66 | 0,77 | 4,44 |
| Bukit Duabelas | Oil Palm | BO4b | Soil | 2013 | 2418 | 13 | 2,16 | 0,84 | 6,60 |
| Bukit Duabelas | Oil Palm | BO5b | Soil | 2013 | 4017 | 5 | 0,96 | 0,55 | 2,24 |
| Bukit Duabelas | Rainforest | BF1b | Soil | 2013 | 1326 | 7 | 1,61 | 0,75 | 4,09 |
| Bukit Duabelas | Rainforest | BF2b | Soil | 2013 | 1755 | 8 | 1,71 | 0,78 | 4,63 |
| Bukit Duabelas | Rainforest | BF3b | Soil | 2013 | 156 | 3 | 1,03 | 0,62 | 2,66 |
| Bukit Duabelas | Rainforest | BF4b | Soil | 2013 | 6201 | 7 | 0,96 | 0,54 | 2,17 |
| Bukit Duabelas | Rubber | BR1b | Soil | 2013 | 2145 | 7 | 1,33 | 0,66 | 3,01 |
| Bukit Duabelas | Rubber | BR2b | Soil | 2013 | 1521 | 5 | 1,28 | 0,67 | 3,12 |
| Bukit Duabelas | Rubber | BR3b | Soil | 2013 | 1014 | 5 | 1,11 | 0,58 | 2,41 |
| Bukit Duabelas | Rubber | BR4b | Soil | 2013 | 507 | 3 | 0,85 | 0,52 | 2,08 |
| Harapan | Jungle Rubber | HJ1b | Soil | 2013 | 1014 | 4 | 1,26 | 0,68 | 3,15 |
| Harapan | Jungle Rubber | HJ3b | Soil | 2013 | 390 | 3 | 0,89 | 0,54 | 2,17 |
| Harapan | Jungle Rubber | HJ4b | Soil | 2013 | 702 | 6 | 1,45 | 0,71 | 3,52 |
| Harapan | Oil Palm | HO1b | Soil | 2013 | 3042 | 7 | 1,28 | 0,63 | 2,76 |
| Harapan | Oil Palm | HO2b | Soil | 2013 | 2262 | 9 | 1,65 | 0,74 | 3,99 |
| Harapan | Oil Palm | HO3b | Soil | 2013 | 468 | 3 | 0,56 | 0,29 | 1,41 |
| Harapan | Rainforest | HF1b | Soil | 2013 | 1833 | 6 | 0,69 | 0,30 | 1,43 |
| Harapan | Rainforest | HF2b | Soil | 2013 | 2223 | 7 | 1,11 | 0,50 | 2,02 |
| Harapan | Rainforest | HF3b | Soil | 2013 | 1560 | 3 | 0,69 | 0,43 | 1,76 |
| Harapan | Rainforest | HF4b | Soil | 2013 | 3042 | 5 | 0,94 | 0,45 | 1,85 |
| Harapan | Rubber | HR1b | Soil | 2013 | 2847 | 6 | 1,35 | 0,67 | 3,08 |
| Harapan | Rubber | HR2b | Soil | 2013 | 1053 | 4 | 0,96 | 0,51 | 2,04 |
| Harapan | Rubber | HR3b | Soil | 2013 | 1170 | 6 | 1,41 | 0,68 | 3,14 |
| Harapan | Rubber | HR4b | Soil | 2013 | 819 | 2 | 0,19 | 0,09 | 1,09 |

**Table S2 Model the effect of systems on ∆^13^C isotopic values**

Fixed effects:

Estimate Std. Error df t value Pr(>|t|)

(Intercept) 2.95355 0.16816 65.67466 17.563 < 2e-16 ***

Oil palm -0.30996 0.24381 155.96151 -1.271 0.205517

Rainforest 0.88519 0.22810 135.10163 3.881 0.000162 ***

Rubber -0.09414 0.21559 153.21799 -0.437 0.662969

Signif. codes: 0 ‘***’ 0.001 ‘**’ 0.01 ‘*’ 0.05 ‘.’ 0.1 ‘ ’ 1

Sum Sq Mean Sq NumDF DenDF F value Pr(>F)

Land.use.System 28.973 9.6576 3 146.05 9.9009 5.532e-06 ***

**Table S3. Significant letter from Tukey-test of the effect of systems on ∆^13^C isotopic values**

Land.use.System emmean SE df lowerCL upperCL group

Oil palm 2.73 0.187 156 2.26 3.20 a

Rubber 2.93 0.155 156 2.54 3.32 a

Jungle rubber 3.01 0.155 156 2.62 3.40 a

Rainforest 3.85 0.158 156 3.45 4.25 b

Confidence level used: 0.95

**Table S4 Model the effect of systems on ∆^15^N isotopic values**

Fixed effects:

Estimate Std. Error df t value Pr(>|t|)

(Intercept) 4.2145 0.5804 61.9174 7.262 7.61e-10 ***

Oil palm -1.2019 0.7341 151.5252 -1.637 0.104

Rainforest -0.1606 0.7130 152.8261 -0.225 0.822

Rubber 0.2538 0.6420 147.1428 0.395 0.693

Signif. codes: 0 ‘***’ 0.001 ‘**’ 0.01 ‘*’ 0.05 ‘.’ 0.1 ‘ ’

Sum Sq Mean Sq NumDF DenDF F value Pr(>F)

Land.use.System 35.934 11.978 3 152.89 1.4592 0.2279

**Table S5. Significant letter from Tukey-test of the effect of systems on ∆^15^N isotopic values**

Land.use.System emmean SE df lower CL upper CL group

Oil palm 4.08 0.597 156 2.58 5.58 a

Jungle rubber 4.29 0.493 156 3.05 5.53 a

Rainforest 4.59 0.504 156 3.32 5.86 a

Rubber 5.03 0.493 156 3.79 6.27 a

Confidence level used: 0.95

**Table S6. ∆^13^C isotopic values of species in rainforest**

Fixed effects:

Estimate Std. Error df t value Pr(>|t|)

(Intercept) 3.6396 0.2654 1 4.2246 13.714 1.35e-09 ***

Isotomiella cf.minor 0.2470 0.2147 2.8048 1.151 0.271

Lepidocyrtus sp.1 -0.1903 0.2277 12.7220 -0.836 0.419

Pararrhopalites sp.1 -0.1412 0.1959 12.4055 -0.721 0.484

Pseudosinella sp.1 0.5791 0.2088 12.7951 2.774 0.016 *

---

Signif. codes: 0 ‘***’ 0.001 ‘**’ 0.01 ‘*’ 0.05 ‘.’ 0.1 ‘ ’ 1

Type III Analysis of Variance Table with Satterthwaite's method

Sum Sq Mean Sq NumDF DenDF F value Pr(>F)

Species 1.7226 0.43064 4 12.539 6.3436 0.005047 **

Species emmean SE df lower.CL upper.CL . group

Lepidocyrtus sp.1 3.45 0.250 12.12 2.69 4.21 a

Pararrhopalites sp.1 3.50 0.234 9.87 2.76 4.24 a

Allacma sp.1 3.64 0.266 14.11 2.85 4.43 ab

Isotomiella cf.minor 3.89 0.240 10.75 3.14 4.63 ab

Pseudosinella sp.1 4.22 0.233 9.79 3.48 4.96 b

**Table S7. ∆^13^C isotopic values of species in jungle rubber**

Fixed effects:

Estimate Std. Error df t value Pr(>|t|)

(Intercept) 3.119551 0.250028 12.850165 12.477 1.49e-08 ***

Callyntrura sp.1 -0.689469 0.264358 16.913427 -2.608 0.0184 *

Folsomides centralis 0.007335 0.216371 16.764134 0.034 0.9734

Homidia cingula 0.315800 0.246299 16.202993 1.282 0.2178

Pseudosinella sp.1 0.024740 0.233050 16.270388 0.106 0.9168

Spyrhoteca sp.1 -0.700629 0.282540 16.649945 -2.480 0.0242 *

---

Signif. codes: 0 ‘***’ 0.001 ‘**’ 0.01 ‘*’ 0.05 ‘.’ 0.1 ‘ ’ 1

Type III Analysis of Variance Table with Satterthwaite's method

Sum Sq Mean Sq NumDF DenDF F value Pr(>F)

Species 2.5235 0.50471 5 16.702 3.6692 0.02001 *

Species emmean SE df lower.CL upper.CL group

Spyrhoteca sp.1 2.42 0.307 19.7 1.52 3.31 ab

Callyntrura sp.1 2.43 0.291 17.9 1.57 3.29 a

Ascocyrtus cinctus 3.12 0.251 13.0 2.34 3.90 ab

Folsomides centralis 3.13 0.240 11.7 2.37 3.89 ab

Pseudosinella sp.1 3.14 0.263 14.7 2.35 3.94 ab

Homidia cingula 3.44 0.280 17.0 2.60 4.27 b

**Table S8. ∆^13^C isotopic values of species in rubber plantations**

Fixed effects:

Estimate Std. Error df t value Pr(>|t|)

(Intercept) 2.2822 0.3767 23.6322 6.059 3.14e-06 ***

Alloscopus tetracanthus 0.9751 0.4256 19.5089 2.291 0.0332 *

Ascocyrtus cinctus 0.6071 0.4268 19.6045 1.423 0.1706

Folsomides centralis 0.7385 0.4127 18.6598 1.789 0.0898 .

Homidia cingula 0.7013 0.4151 18.9387 1.689 0.1075

Pseudosinella sp.1 0.9245 0.3890 18.9302 2.377 0.0282 *

---

Signif. codes: 0 ‘***’ 0.001 ‘**’ 0.01 ‘*’ 0.05 ‘.’ 0.1 ‘ ’ 1

Type III Analysis of Variance Table with Satterthwaite's method

Sum Sq Mean Sq NumDF DenDF F value Pr(>F)

Species 2.0113 0.40226 5 18.611 1.3968 0.2708

Species emmean SE df lower.CL upper.CL group

Acrocyrtus sp.1 2.28 0.383 23.6 1.18 3.38 a

Ascocyrtus cinctus 2.89 0.311 20.2 1.98 3.80 a

Homidia cingula 2.98 0.311 20.3 2.08 3.89 a

Folsomides centralis 3.02 0.311 20.3 2.12 3.93 a

Pseudosinella sp.1 3.21 0.271 16.8 2.40 4.01 a

Alloscopus tetracanthus 3.26 0.311 20.2 2.35 4.16 a

**Table S9. ∆^13^C isotopic values of species in oil palm plantations**

| Fixed effects:  Estimate Std. Error df t value Pr(>\|t\|)  (Intercept) 2.9032 0.6120 9.2615 4.744 0.000973 ***  Isotomiella cf.minor -0.3096 0.4321 8.8038 -0.717 0.492224  Pseudosinella sp.1 0.2398 0.3106 8.2876 0.772 0.461456  ---  Signif. codes: 0 ‘***’ 0.001 ‘**’ 0.01 ‘*’ 0.05 ‘.’ 0.1 ‘ ’ 1  Type III Analysis of Variance Table with Satterthwaite's method  Sum Sq Mean Sq NumDF DenDF F value Pr(>F)  Species 0.62334 0.31167 2 8.59 1.1034 0.3745 |
| --- |
| Species emmean SE df lower.CL upper.CL group  Isotomiella cf.minor 2.59 0.634 10.01 0.781 4.41 a  Folsomides centralis 2.90 0.613 9.11 1.115 4.69 a  Pseudosinella sp.1 3.14 0.584 7.70 1.371 4.91 a |
| \|  \| \| --- \| |

**Table S10. ∆^15^N isotopic values of species in rainforest**

Fixed effects:

Estimate Std. Error df t value Pr(>|t|)

(Intercept) 0.5851 1.8664 18.9925 0.313 0.75734

Isotomiella cf.minor 5.0897 2.1449 14.7976 2.373 0.03165 *

Lepidocyrtus sp.1 3.8540 2.2794 14.9412 1.691 0.11162

Pararrhopalites sp.1 2.4453 2.0135 13.3735 1.214 0.24558

Pseudosinella sp.1 8.2953 2.0858 14.8565 3.977 0.00124 **

---

Signif. codes: 0 ‘***’ 0.001 ‘**’ 0.01 ‘*’ 0.05 ‘.’ 0.1 ‘ ’ 1

Type III Analysis of Variance Table with Satterthwaite's method

Sum Sq Mean Sq NumDF DenDF F value Pr(>F)

Species 149.77 37.442 4 13.889 4.9994 0.01041 *

Species emmean SE df lower.CL upper.CL group

Allacma sp.1 0.585 1.93 19.0 -4.906 6.08 a

Pararrhopalites sp.1 3.030 1.41 17.0 -1.041 7.10 a

Lepidocyrtus sp.1 4.439 1.69 18.8 -0.377 9.26 ab

Isotomiella cf.minor 5.675 1.52 18.1 1.315 10.03 ab

Pseudosinella sp.1 8.880 1.40 17.1 4.838 12.92 b

**Table S11. ∆^15^N isotopic values of species in jungle rubber**

Fixed effects:

Estimate Std. Error df t value Pr(>|t|)

(Intercept) 4.7948 1.3031 18.6083 3.680 0.00164 **

Callyntrura sp.1 -5.8402 1.7536 18.5634 -3.330 0.00360 **

Folsomides centralis 0.4686 1.4440 17.6247 0.325 0.74937

Homidia cingula -1.1271 1.6579 16.9173 -0.680 0.50582

Pseudosinella sp.1 2.6967 1.5664 17.0943 1.722 0.10319

Spyrhoteca sp.1 -0.6216 1.8844 17.9573 -0.330 0.74531

---

Signif. codes: 0 ‘***’ 0.001 ‘**’ 0.01 ‘*’ 0.05 ‘.’ 0.1 ‘ ’ 1

Type III Analysis of Variance Table with Satterthwaite's method

Sum Sq Mean Sq NumDF DenDF F value Pr(>F)

Species 148.85 29.77 5 17.886 4.7191 0.006342 **

Species emmean SE df lower.CL upper.CL group

Callyntrura sp.1 -1.05 1.64 21.7 -5.797 3.71 a

Homidia cingula 3.67 1.56 21.9 -0.835 8.17 ab

Spyrhoteca sp.1 4.17 1.77 22.8 -0.914 9.26 ab

Ascocyrtus cinctus 4.79 1.32 18.6 0.926 8.66 b

Folsomides centralis 5.26 1.23 17.2 1.615 8.91 b

Pseudosinella sp.1 7.49 1.42 20.3 3.358 11.63 b

**Table S12. ∆^15^N isotopic values of species in rubber plantations**

Fixed effects:

Estimate Std. Error df t value Pr(>|t|)

(Intercept) 8.249 1.306 24.000 6.315 1.58e-06 ***

Alloscopus tetracanthus -2.920 1.577 21.025 -1.852 0.0781 .

Ascocyrtus cinctus -4.205 1.579 21.161 -2.662 0.0145 *

Folsomides centralis -3.798 1.544 19.753 -2.460 0.0232 *

Homidia cingula -4.357 1.548 20.087 -2.814 0.0107 *

Pseudosinella sp.1 -1.799 1.453 19.802 -1.238 0.2300

---

Signif. codes: 0 ‘***’ 0.001 ‘**’ 0.01 ‘*’ 0.05 ‘.’ 0.1 ‘ ’ 1

Type III Analysis of Variance Table with Satterthwaite's method

Sum Sq Mean Sq NumDF DenDF F value Pr(>F)

Species 50.378 10.076 5 19.425 2.4309 0.07194 .

Species emmean SE df lower.CL upper.CL group

Homidia cingula 3.89 1.050 23.1 0.874 6.91 a

Ascocyrtus cinctus 4.04 1.050 23.1 1.026 7.06 a

Folsomides centralis 4.45 1.048 23.1 1.437 7.47 a

Alloscopus tetracanthus 5.33 1.049 23.1 2.314 8.34 a

Pseudosinella sp.1 6.45 0.887 21.5 3.881 9.02 a

Acrocyrtus sp.1 8.25 1.344 24.0 4.396 12.10 a

**Table S13. ∆^15^N isotopic values of species in oil palm plantations**

Fixed effects:

Estimate Std. Error df t value Pr(>|t|)

(Intercept) 3.5453 0.4383 11.6928 8.089 3.97e-06 ***

Isotomiella cf.minor 0.4392 0.4349 9.8314 1.010 0.336792

Pseudosinella sp.1 2.0362 0.3194 8.6821 6.375 0.000151 ***

---

Signif. codes: 0 ‘***’ 0.001 ‘**’ 0.01 ‘*’ 0.05 ‘.’ 0.1 ‘ ’ 1

Type III Analysis of Variance Table with Satterthwaite's method

Sum Sq Mean Sq NumDF DenDF F value Pr(>F)

Species 14.248 7.1238 2 9.3161 23.589 0.0002259 ***

Species emmean SE df lower.CL upper.CL group

Folsomides centralis 3.55 0.443 11.4 2.31 4.78 a

Isotomiella cf.minor 3.98 0.471 12.8 2.69 5.28 a

Pseudosinella sp.1 5.58 0.398 8.6 2.41 6.76 b

**Supplementary Material Figure 1**

Isotopic niche of Collembola species in different land-use systems


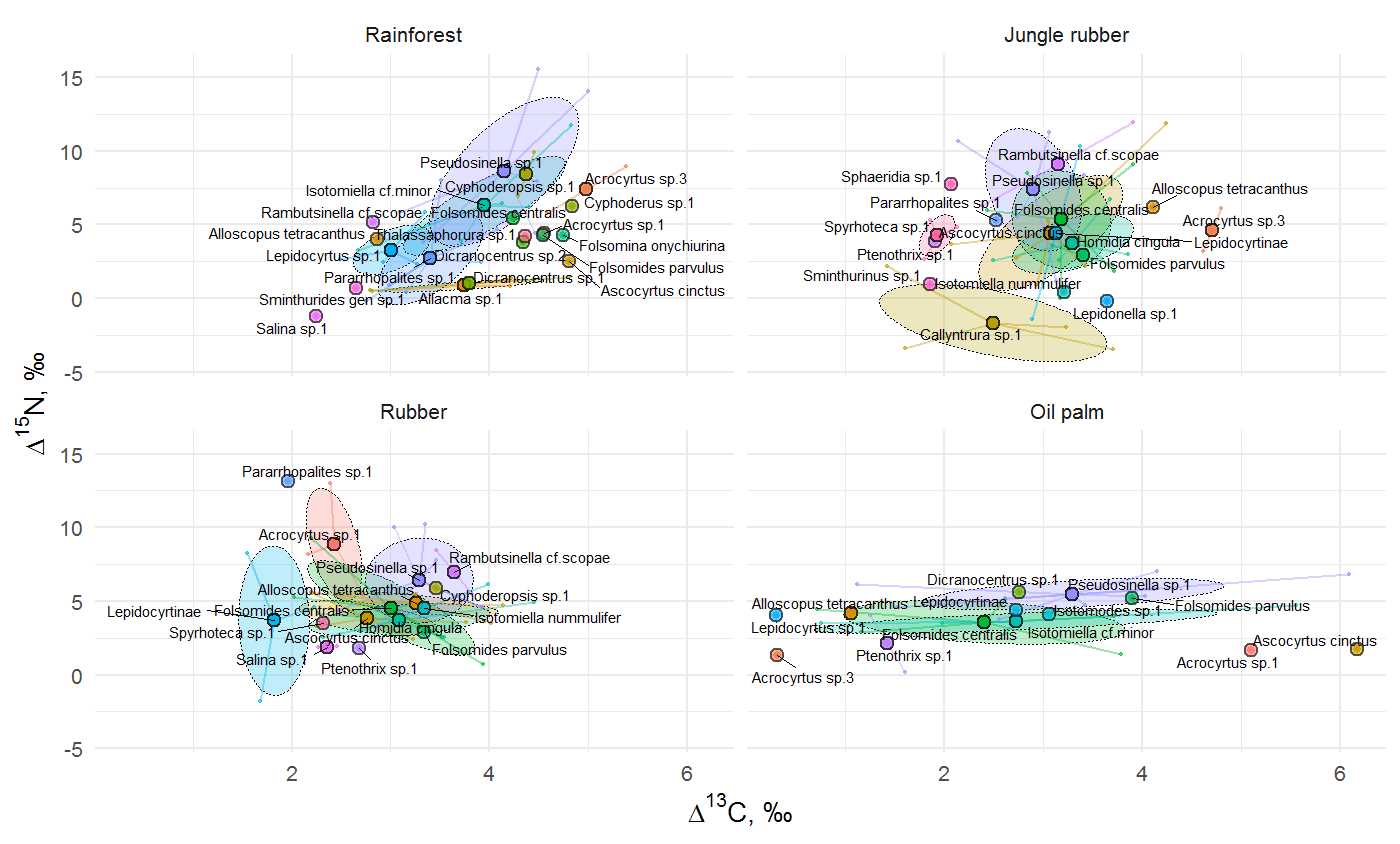


**Figure 1 .** Isotopic niche of Collembola species in different land-use systems (rainforest, jungle rubber, rubber and oil palm plantations). Ellipses denote 60% confidence intervals, different species are shown with colors. Large colored dots represent means of land-use systems, small points represent individual measurements.
